# Development and external validation of an interpretable early prediction model for acute kidney injury using TabPFN and routine admission data: a retrospective cohort study

**DOI:** 10.1186/s12911-026-03561-7

**Published:** 2026-05-12

**Authors:** Zheng Xu, Chenyu Li, Chen Guan, Lingyu Xu, Xinyuan Wang, Siqi Jiang, Ningxin Zhang, Minghao Gu, Yanlu Xin, Yan Xu

**Affiliations:** Department of Nephrology, The Affiliated Hospital of Qingdao University, No. 16 Jiangsu Road, Shinan District, Qingdao, Shandong Province China

**Keywords:** Acute kidney injury, Tabular Prior-data Fitted Network, electronic health record system, Vulnerability–stress interaction, SHAP

## Abstract

**Background:**

Acute kidney injury (AKI) is a common and serious complication among hospitalized patients, and early risk stratification remains challenging due to heterogeneous clinical data and limited interpretability of existing prediction models. Foundation models designed for tabular data may enable accurate prediction while preserving interpretability, but their application in early AKI risk assessment has not been fully explored.

**Objective:**

To develop and externally validate an interpretable early prediction model for AKI using the Tabular Prior-data Fitted Network (TabPFN) based on routinely available admission data.

**Methods:**

In this retrospective cohort study, predictors were restricted to clinical variables recorded within the 24 h prior to hospital admission, and this temporal definition was applied consistently across both the internal cohort and the external MIMIC-IV validation dataset. Time zero was defined as the admission time. Baseline serum creatinine (SCr) was defined as the first creatinine measurement at admission. The primary outcome was in-hospital AKI, defined according to KDIGO SCr criteria as a subsequent rise in creatinine relative to this admission baseline at any time during the index hospitalization. A total of 44,324 patients were included in the development cohort. TabPFN was trained on a stratified subsample and evaluated on a held-out internal test set, and benchmarked against seven conventional machine-learning models. Missing data were handled using multivariate imputation, and model interpretation was performed using SHAP-based attribution analyses. External validation was conducted in the MIMIC-IV database following predefined inclusion criteria and feature harmonization.

**Results:**

In the internal test set, TabPFN achieved an AUROC of 0.953, outperforming comparator models. External validation demonstrated robust discrimination with an AUROC of 0.859. Calibration analyses indicated good agreement between predicted and observed risks. Attribution analyses identified baseline renal function and acute illness markers as major contributors to model-attributed AKI risk, with heterogeneous association patterns across patient subgroups.

**Conclusions:**

Using routinely available pre-admission data, TabPFN enabled accurate early prediction of in-hospital AKI and provided interpretable risk attribution patterns. These findings suggest potential utility for early risk stratification; however, results are observational and hypothesis-generating, and prospective validation is required before clinical deployment.

**Supplementary Information:**

The online version contains supplementary material available at 10.1186/s12911-026-03561-7.

## Highlights

TabPFN enabled early prediction of in-hospital AKI using routinely available admission data.

External validation in MIMIC-IV demonstrated preserved discrimination under cross-cohort dataset shift.

SHAP analyses identified heterogeneous model-attributed risk patterns across baseline renal function subgroups.

**Supplementary Information:**

The online version contains supplementary material available at 10.1186/s12911-026-03561-7.

## Introduction

Acute kidney injury (AKI) is a common and serious clinical syndrome characterized by a rapid decline in kidney function and is associated with increased mortality, prolonged hospitalization, and long-term progression to chronic kidney disease [1, 2]. Recent epidemiological data indicate that AKI affects approximately 10–15% of hospitalized patients and more than half of critically ill individuals, representing a major global health burden and a key target for early intervention strategies [1, 3]. Current diagnostic criteria primarily rely on serum creatinine (SCr) changes, which typically occur after structural kidney injury has already developed, thereby limiting opportunities for timely prevention and early clinical decision-making [2, 4].

Recent advances in machine learning have improved AKI prediction performance using electronic health record (EHR) data, with several studies demonstrating improved discrimination compared with traditional statistical approaches [5–7]. However, many existing models rely on dynamic longitudinal trajectories during hospitalization and often function as complex black-box systems, which may limit interpretability and early decision support at the time of admission [8]. In addition, external validation and robustness across heterogeneous populations remain major challenges in clinical artificial intelligence research [9].

Tabular Prior-data Fitted Networks (TabPFN) represent a recently developed class of foundation models designed for tabular data, demonstrating strong predictive performance without extensive task-specific hyperparameter tuning [10]. Despite growing interest in foundation models, their application in early admission-based AKI prediction has not been systematically evaluated. Therefore, the present study aimed to develop and externally validate an interpretable early prediction model for in-hospital AKI using only clinical data available within the 24 h prior to hospital admission. By restricting predictors to pre-admission information and defining admission as time zero, this study evaluates early risk stratification within a temporally rigorous framework and characterizes model-attributed risk patterns as statistical associations.

## Method

### Data collection

This study was conducted using data extracted from the EHR system of a university affiliated hospital, a large multi-campus medical center in China. This was a retrospective cohort study. Time zero was defined as the hospital admission time. Predictors were restricted to clinical variables recorded within the 24 h prior to admission. Pre-admission data referred to laboratory tests and vital signs documented within 24 h before the admission time in the EHR system, including emergency department evaluations and pre-admission assessments. Only data temporally preceding admission were used to ensure separation between predictor information and outcome assessment.

### Outcome definition

AKI was defined according to the Kidney Disease: Improving Global Outcomes (KDIGO) 2012 serum creatinine (SCr) criteria. Baseline SCr was defined as the first creatinine measurement at admission. In-hospital AKI was defined as a subsequent rise in SCr relative to this admission baseline, meeting KDIGO criteria: an increase in SCr of ≥ 26.5 µmol/L (0.3 mg/dL) within 48 h, or an increase to ≥ 1.5 times baseline presumed to have occurred within 7 days [4]. Because urine output measurements were not continuously available or reliably documented in both the internal electronic health record dataset and the MIMIC-IV cohort, and given variability in recording practices across healthcare institutions, the KDIGO urine output criterion was not applied. To ensure consistent outcome assessment across cohorts, AKI was therefore defined solely based on changes in serum creatinine.

### Model development and interpretation

Before model development, prespecified inclusion and exclusion criteria were applied to define the analytic cohort. Predictors were restricted to variables recorded within the 24 h prior to admission. The dataset was first split into a training set (80%) and a held-out test set (20%) using stratified sampling to preserve AKI event proportions. To avoid information leakage, variable-level missingness was assessed using the training set only. Predictors with more than 15% missing values in the training data were excluded, and the resulting feature set was applied consistently to the test set and external validation cohort to ensure identical predictor availability. Missing values were subsequently handled using Multivariate Imputation by Chained Equations [11] (MICE). Imputation models were fitted exclusively on the training data and then applied to the test set. For external validation, imputation was performed separately using the same predefined feature set. TabPFN was originally designed for small- to medium-sized tabular datasets and has a practical upper input limit of approximately 10,000 samples in its current implementation, a stratified subset of 10,000 patients from the training cohort was used for TabPFN model fitting. To benchmark TabPFN, we implemented seven baseline machine learning models using the scikit-learn [12] and gradient boosting libraries: Logistic Regression (LR), Support Vector Machine (SVM), Random Forest (RF), Gradient Boosting Machine (GBM), Extreme Gradient Boosting (XGBoost) [13], CatBoost [14], Light GBM (LightGBM) [15]. TabPFN and all comparator models were trained using the same training–test split, identical feature sets, and identical imputed datasets to ensure consistent evaluation.

SHAP (SHapley Additive exPlanations) values were calculated to estimate feature contributions to predicted risk. These analyses characterize model-attributed associations and do not imply causal relationships. For TabPFN, we utilized the official *tabpfn_extensions.interpretability* module, which supports SHAP-like attribution via permutation-based estimation. For cross-model feature importance comparison, we applied the official SHAP Python package (version 0.50.0) to seven models.

### External validation

To evaluate the generalizability of our model, we performed external validation using MIMIC-IV (2008–2019): a comprehensive medical database comprising clinical records of hundreds of thousands of patients, commonly used in healthcare AI and informatics research [16, 17]. Among the 53,150 hospitalized patients identified in MIMIC-IV, individuals with fewer than two serum creatinine (SCr) measurements (*n* = 4,081), those receiving renal replacement therapy or with renal transplantation (*n* = 6,211), and those with hospitalization duration < 48 h (*n* = 1,898) were excluded according to the same criteria applied to the development cohort. After these shared exclusions, 40,960 patients remained eligible for external validation. Since not all predictors available in the internal cohort were present in MIMIC-IV, external validation was conducted using the intersection of features shared between the two cohorts. To ensure comparability, the model was re-trained in the internal training set using this harmonized feature set and then evaluated on the internal test set and the external cohort under an identical preprocessing and imputation pipeline. To assess the robustness of this feature-missingness threshold, we conducted sensitivity analyses using alternative missing-value cutoffs. For each threshold, we evaluated the resulting sample size, AKI incidence, and model discrimination performance. This analysis allowed us to examine the stability of external validation results under varying degrees of feature completeness.

### Statistical analysis

Continuous variables were presented as mean ± standard deviation for normally distributed data or median with interquartile range (IQR) for non-normally distributed data. Between-group comparisons were performed using the independent-samples t-test for normally distributed variables and the Wilcoxon rank-sum test for nonparametric data. Categorical variables were expressed as frequencies and percentages and compared using the chi-square test or Fisher’s exact test where appropriate.The performance of predictive models was assessed on the held-out test set. Discrimination was evaluated using the area under the receiver operating characteristic curve (AUROC) and the area under the precision–recall curve (AUPRC). AUPRC was calculated as the area under the precision–recall curve (equivalent to the average precision score). 95% confidence intervals (CIs) for AUROC and AUPRC were estimated using stratified bootstrap resampling with 1,000 iterations. Additional classification metrics, including accuracy, sensitivity, specificity, precision, recall, and F1-score, were calculated at the optimal classification threshold. Calibration performance was assessed using calibration plots and quantified using the Brier score. To evaluate the potential clinical utility of model-guided decision-making, decision curve analysis (DCA) was performed to estimate net benefit across a range of threshold probabilities, comparing the model against “treat-all” and “treat-none” strategies.

For model interpretation, feature importance was examined using SHAP values. Cross-model consistency of feature rankings was evaluated using Spearman’s rank correlation coefficient. Subgroups analyses were conducted to explore variations in mean SHAP values across eGFR subgroups. Threshold sensitivity analyses were performed to assess the stability of key performance metrics across different classification cutoffs. All statistical analyses were performed using Python (version 3.12.7).

## Results

### Baseline characteristics and outcomes

**Fig. 1:**
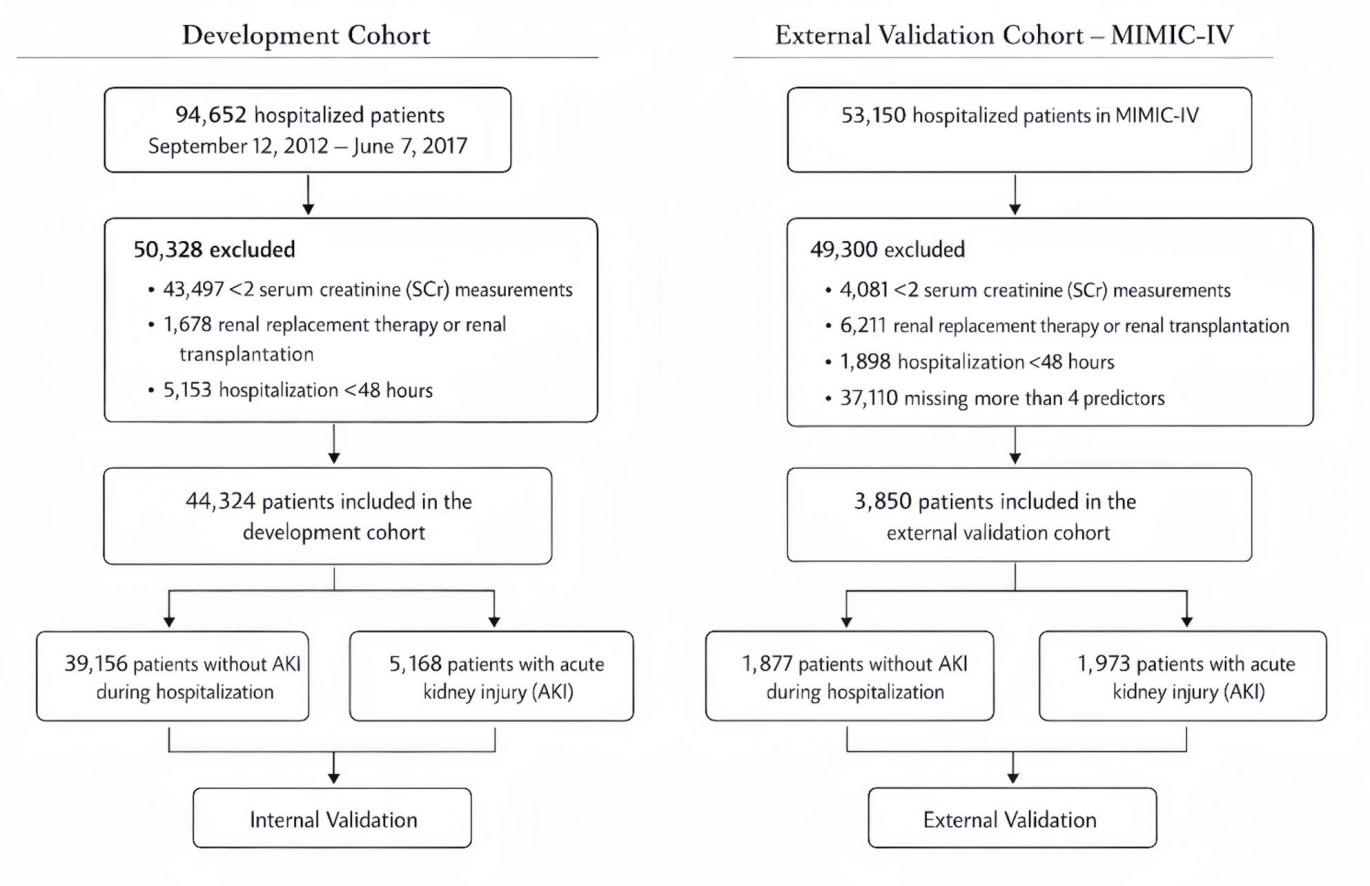
Flowchart of patient selection. A total of 94,652 hospitalized patients from September 12, 2012 to June 7, 2017 were screened. After applying exclusion criteria, 44,324 patients were included in the analysis, including 5,168 patients with AKI and 39,156 AKI-free patients. The dataset was randomly split into a training cohort (80%) and a test cohort (20%)

From an initial 94,652 screened patients, 44,324 were enrolled for final analysis (Fig. 1). The study cohort had an average age of 60 years and consisted predominantly of males (59.53%, *n* = 26,385). The average baseline eGFR was 96.71mL/min/1.73 m². During hospitalization, 5,168 patients (11.68%) developed AKI, and a total of 1,467 patients died. The median length of stay was 17 days. The mortality rate was significantly higher in the AKI group (17.37%, *n* = 899) than in the AKI-free group (1.45%, *n* = 568). Compared with the AKI-free group, the clinical condition of AKI patients at admission was worse, as reflected by significant differences in key laboratory indicators (Table 1; the complete baseline characteristics are available in Supplementary Table S1).

**Table 1:** The partial baseline characteristics of the current cohort

Variable	AKI-free		AKI		|t/Z/χ²|		*p*-value
Demographics							
Male (n/%)	23,434 (59.8%)		2953 (57.1%)		13.782		< 0.001
Age (year)	63 (52–72)		63 (49–73)		2.318		0.02
Smoke (n/%)	13,568 (34.7%)		1593 (30.8%)		29.539		< 0.001
Drink (n/%)	10,948 (28.0%)		1310 (25.3%)		15.434		< 0.001
BMI (kg/m²)	24.20 (21.60–26.10)		24.20 (21.50–26.60)		3.496		< 0.001
Operation (n/%)	14,414 (36.8%)		1225 (23.7%)		342.968		< 0.001
SBP (mmHg)	129 (116–141)		130 (115–146)		4.312		< 0.001
DBP (mmHg)	78 (70–85)		78 (68–87)		0.432		0.665
Laboratory tests							
Scr (µmol/L)	80.00 (66.20–94.00)		69.00 (46.18-111.45)		15.915		< 0.001
eGFR (mL/min/1.73 m²)	82.10 (68.00-102.20)		98.20 (55.30-147.83)		14.455		< 0.001
Outcomes							
LOS (day)	17 (14–24)		13 (7–25)		31.636		< 0.001
Death (n/%)	568 (1.5%)		899 (17.4%)		3614.562		< 0.001

Continuous variables are expressed as mean ± standard deviation or median (interquartile range), and categorical variables as number (percentage). P-values were calculated for comparisons between the AKI and AKI-free groups.*BMI: Body Mass Index; SBP: Systolic Blood Pressure; DBP: Diastolic Blood Pressure; Scr: Serum Creatinine; LOS: Length of Stay

### Model interpretations

**Fig. 2:**
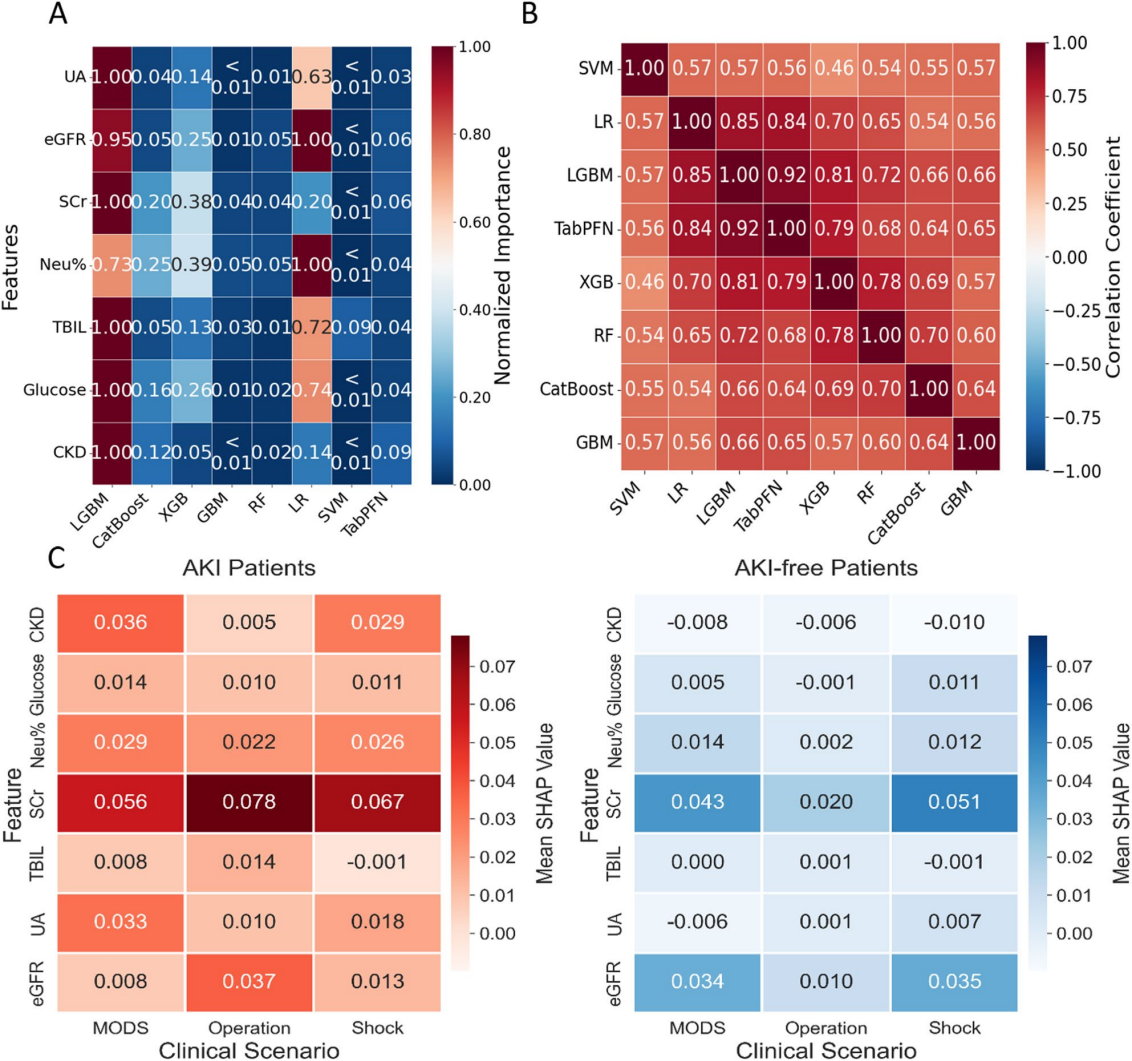
Feature importance, model correlations, and SHAP-based vulnerability–stress interactions. (**A**) Normalized feature importance across multiple machine-learning models. (**B**) Correlation of feature-importance profiles among models. (**C**) Vulnerability–stress SHAP interaction patterns in AKI and AKI-free patients

SHAP analysis was conducted across all eight models to identify features contributing to predicted AKI risk. To address heterogeneity in feature rankings, a cross-model voting strategy was applied: features appearing in at least 4 of 8 models’ Top 10 lists were defined as consensus features. This approach identified 7 consensus predictors shared across models. Inter-model correlation analysis demonstrated moderate to high agreement among certain algorithms (Spearman’s ρ: 0.42–0.92), indicating variability in feature utilization patterns across model classes (Fig. 2A–B). Subgroups SHAP analyses stratified by baseline eGFR (> 90, 60–90, < 60 mL/min/1.73 m²) revealed differences in model-attributed risk contributions across renal function subgroups. For example, shock showed higher mean SHAP values in patients with reduced baseline kidney function, whereas surgery exhibited relatively modest contributions across groups. Multiple organ dysfunction syndrome (MODS) demonstrated consistently high SHAP contributions (Supplementary Figure S1). Exploratory interaction analyses suggested variation in joint SHAP contributions between selected vulnerability and stress-related variables (Supplementary Figure S1C), These findings describe model-attributed patterns. In addition, SHAP value distributions for representative consensus features (eGFR, UA, Neu%, and SCr) are presented in (Supplementary Figure S2), illustrating differences in SHAP magnitude between AKI and AKI-free groups. These distributions further characterize model-attributed contribution patterns at the individual feature level.

### Model performance

**Fig. 3:**
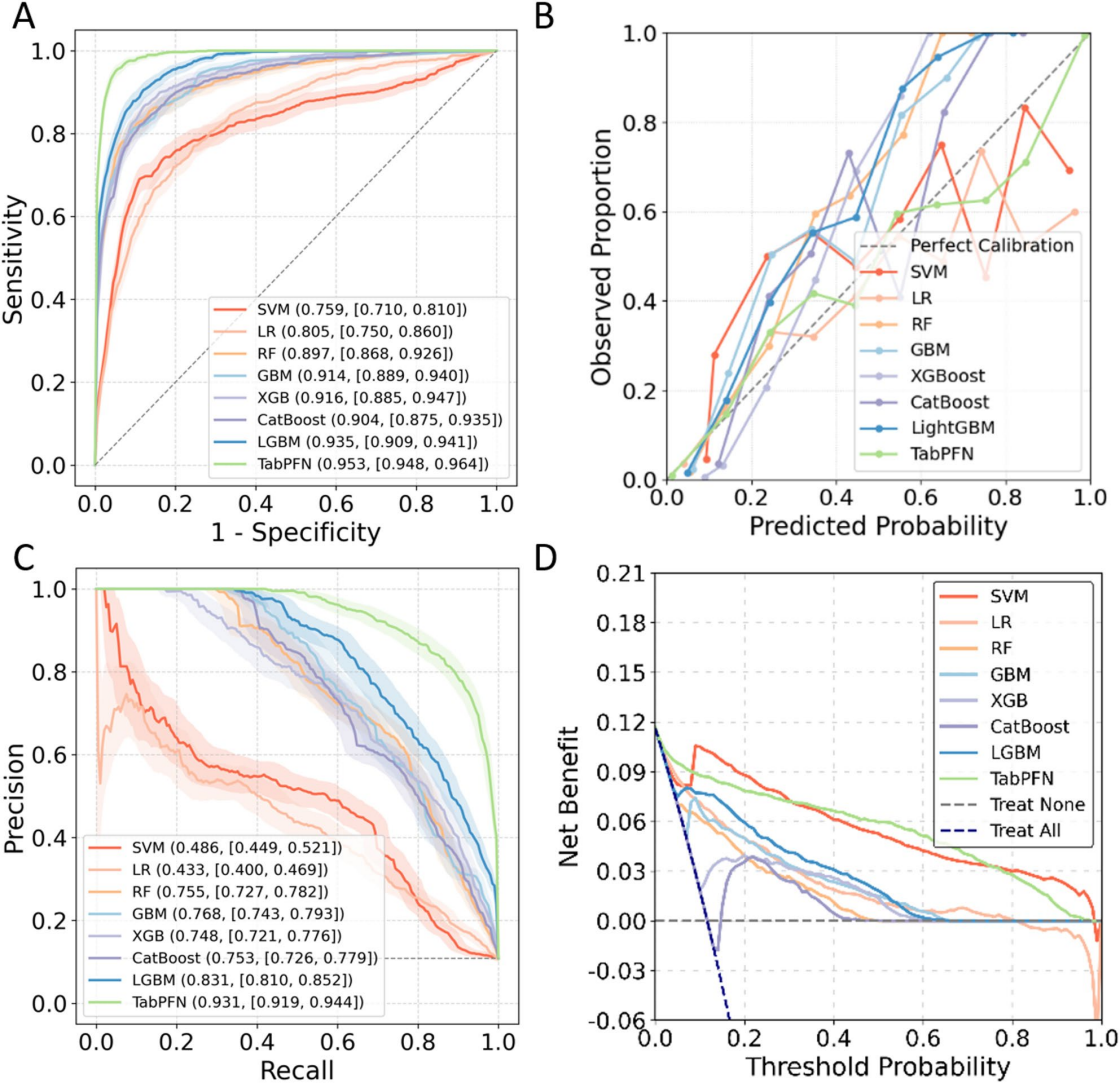
Performance comparison and calibration of predictive models for AKI. (**A**) ROC curves of eight models in the internal cohort (**B**) Calibration curve of eight models in the internal cohort (**C**) PRC curves of eight models in the internal cohort (**D**) Decision curve analysis of eight models in the internal cohort

On the held-out test set, TabPFN achieved the strongest discrimination among all evaluated models, as reflected by the highest AUROC and AUPRC (Table 2; Fig. 3A and C). Tree-based ensemble models demonstrated competitive but slightly lower performance, whereas linear and kernel-based models showed comparatively reduced discrimination. Calibration analysis indicated good agreement between predicted and observed risks for TabPFN, with calibration slope close to unity and intercept near zero (Fig. 3B). Additional calibration and probability estimation metrics, including Brier score, log loss, expected calibration error (ECE), and maximum calibration error (MCE), are provided in Supplementary Table S4. In contrast, several boosting models exhibited steeper slopes, suggesting a tendency toward overconfident probability estimates. Decision curve analysis demonstrated that TabPFN provided consistently higher net benefit across a broad range of threshold probabilities compared with alternative models as well as “treat-all” and “treat-none” strategies (Fig. 3D). In addition, a parsimonious logistic regression model incorporating routinely available admission variables (SCr, platelet count, white blood cell count, sex, age, systolic and diastolic blood pressure, and total bilirubin) was developed to represent a simplified clinical risk assessment scenario. Compared with this baseline clinical model, TabPFN achieved superior discrimination and demonstrated consistently higher net benefit across clinically relevant threshold probabilities (Supplementary Figure S3).

**Table 2:** Performance of eight models for predicting AKI

Model	AUROC	AUPRC	F1-score	Precision	Recall	Accuracy
**Training set**						
SVM	0.798 (0.749, 0.849)	0.526 (0.491–0.562)	0.086 (0.038, 0.138)	0.927 (0.840, 1.000)	0.045 (0.000, 0.081)	0.888 (0.884, 0.892)
LR	0.815 (0.740, 0.880)	0.458 (0.423–0.497)	0.269 (0.200, 0.338)	0.625 (0.505, 0.745)	0.172 (0.100, 0.244)	0.892 (0.886, 0.900)
RF	0.906 (0.894, 0.918)	0.761 (0.734–0.788)	0.490 (0.457, 0.531)	0.924 (0.876, 0.972)	0.334 (0.266, 0.404)	0.919 (0.916, 0.922)
GBM	0.924 (0.912, 0.938)	0.780 (0.755–0.805)	0.501 (0.463, 0.540)	0.980 (0.957, 1.000)	0.336 (0.264, 0.406)	0.922 (0.919, 0.925)
XGB	0.928 (0.909, 0.947)	0.761 (0.735–0.788)	0.418 (0.375, 0.461)	0.948 (0.908, 0.988)	0.268 (0.203, 0.333)	0.913 (0.907, 0.919)
CatBoost	0.906 (0.887, 0.925)	0.757 (0.730–0.783)	0.490 (0.450, 0.542)	0.932 (0.891, 0.973)	0.332 (0.267, 0.403)	0.919 (0.916, 0.922)
LGBM	0.953 (0.946, 0.960)	0.848 (0.829–0.868)	0.519 (0.470, 0.556)	0.982 (0.963, 1.000)	0.353 (0.283, 0.425)	0.924 (0.919, 0.929)
**TabPFN**	**0.975 (0.970**,** 0.982)**	**0.953 (0.943–0.963)**	**0.745 (0.710**,** 0.790)**	**0.901 (0.865**,** 0.945)**	**0.636 (0.565**,** 0.707)**	**0.949 (0.944**,** 0.954)**
**Test set**						
SVM	0.759 (0.710, 0.810)	0.486 (0.449–0.521)	0.048 (0.000, 0.100)	0.730 (0.600, 0.860)	0.025 (0.000, 0.057)	0.885 (0.882, 0.888)
LR	0.805 (0.750, 0.860)	0.433 (0.400–0.469)	0.252 (0.180, 0.330)	0.556 (0.400, 0.710)	0.163 (0.070, 0.260)	0.887 (0.883, 0.891)
RF	0.897 (0.868, 0.926)	0.755 (0.727–0.782)	0.473 (0.400, 0.540)	0.927 (0.890, 1.000)	0.318 (0.250, 0.390)	0.918 (0.915, 0.921)
GBM	0.914 (0.889, 0.940)	0.768 (0.743–0.793)	0.468 (0.390, 0.546)	0.961 (0.930, 0.992)	0.310 (0.230, 0.390)	0.918 (0.915, 0.921)
XGB	0.916 (0.885, 0.947)	0.748 (0.721–0.776)	0.384 (0.310, 0.458)	0.945 (0.900, 0.990)	0.242 (0.170, 0.314)	0.910 (0.907, 0.913)
CatBoost	0.904 (0.875, 0.935)	0.753 (0.726–0.779)	0.473 (0.390, 0.550)	0.934 (0.890, 0.979)	0.318 (0.250, 0.386)	0.918 (0.915, 0.921)
LGBM	0.935 (0.909, 0.961)	0.831 (0.810–0.852)	0.487 (0.400, 0.554)	0.960 (0.930, 0.990)	0.327 (0.250, 0.404)	0.920 (0.917, 0.923)
**TabPFN**	**0.953 (0.948**,** 0.964)**	**0.931 (0.919–0.944)**	**0.647 (0.600**,** 0.740)**	**0.807 (0.740**,** 0.880)**	**0.542 (0.450**,** 0.630)**	**0.931 (0.928**,** 0.934)**

Model abbreviations: SVM (Support Vector Machine); LR (Logistic Regression); RF (Random Forest); GBM (Gradient Boosting Machine); XGB (Extreme Gradient Boosting); CatBoost (Categorical Boosting); LGBM (Light Gradient Boosting Machine); TabPFN (Tabular Prior-data Fitted Network)

### Stability and generalization analysis

To assess model stability at the predefined 10,000-sample setting, we performed 50 independent repetitions using stratified resampling. Across these repeated experiments, AUROC showed minimal variability (mean 0.956, SD 0.014), supporting the stability of the model under repeated resampling. In addition, we explored performance under different train/test split ratios (50/50 to 90/10) and varying training sample sizes (2,000–10,000 samples). Across these configurations, AUROC values showed only modest variation, suggesting that model performance was not materially sensitive to partitioning strategy or moderate changes in sample size (Supplementary Figure S4, Table S2).

### External validation

In the primary analysis, patients with more than four missing predictor variables were excluded to ensure adequate feature completeness, resulting in a cohort of 3,850 patients. However, recognizing that this threshold excluded a substantial proportion of otherwise eligible patients, we performed additional sensitivity analyses using alternative row-level missingness criteria, including no row filtering (*n* = 40,960) and ≤ 7 missing features (*n* = 18,788). As expected, sample size and AKI incidence varied across thresholds (Supplementary Table S7). Despite these differences, model discrimination remained relatively consistent. The ≤ 4-missing threshold yielded the highest AUROC, followed by the ≤ 7 threshold, whereas performance under no row filtering was slightly lower (Supplementary Figure S5).

## Discussion

This study developed and externally validated an early prediction model for in-hospital acute kidney injury (AKI) using routinely available clinical variables recorded within the 24 h prior to admission. By defining admission as time zero and restricting predictors to pre-admission data, we evaluated AKI risk stratification within a strictly temporally separated framework. In internal testing, the TabPFN model demonstrated strong discrimination (AUROC 0.953; AUPRC 0.931), outperforming conventional machine-learning comparators trained under identical data conditions. External validation in the MIMIC-IV cohort showed a decrease in discrimination (AUROC 0.859), yet performance remained within a clinically acceptable range in a geographically and demographically distinct population. Calibration analysis indicated reasonable agreement between predicted and observed risk in both cohorts. Collectively, these findings suggest that foundation models designed for tabular data can achieve robust early AKI risk prediction using static admission-level information, while maintaining interpretable model-attributed risk patterns.

Over the past decade, predictive modeling for AKI has progressed from conventional regression-based and ensemble machine-learning methods to increasingly complex deep-learning architectures [18–20]. Traditional approaches such as logistic regression, random forests, and gradient boosting models have demonstrated moderate discrimination across heterogeneous inpatient cohorts, typically reporting AUROC values in the range of 0.70–0.85 depending on population and prediction horizon [18–20]. More recently, deep neural networks, convolutional models, and transformer-based frameworks have been applied to large-scale EHR datasets, often leveraging longitudinal trajectories or high-dimensional feature representations to achieve improved discrimination [7, 21–23]. While these models have shown promising performance, they frequently require extensive hyperparameter tuning, large training samples, and dynamic time-series inputs, which may limit portability across institutions and increase implementation complexity [19, 21, 22]. In contrast, relatively fewer studies have focused on strictly admission-based risk prediction using only baseline clinical information available prior to or at the time of hospital entry. Early risk stratification at admission poses a distinct methodological challenge, as it relies on static data and cannot incorporate evolving laboratory trends or in-hospital exposures. Foundation models designed specifically for tabular data, such as TabPFN [10], offer an alternative paradigm by embedding prior knowledge from large-scale synthetic tasks and enabling strong predictive performance with minimal task-specific optimization. Our findings extend prior AKI prediction research by demonstrating that such a tabular foundation model can achieve competitive discrimination under a temporally rigorous admission-based framework, while maintaining stable performance across repeated resampling and external validation. Rather than replacing existing dynamic models, this approach may complement them by providing early baseline risk estimation before in-hospital physiological deterioration becomes apparent.

Beyond discrimination performance, we examined model-attributed feature contributions using SHAP-based analyses to better understand how the model allocated predicted risk across clinical variables. It is important to emphasize that SHAP values reflect internal model associations rather than biological mechanisms or causal relationships [8]. Variables such as shock, multiple organ dysfunction syndrome (MODS), bilirubin, and inflammatory markers likely represent composite indicators of overall illness severity and may function as mediators or proxies of physiological instability rather than independent causal drivers of AKI. In this context, the attribution patterns may help contextualize model predictions by highlighting physiological domains associated with elevated AKI risk. When the model identifies a patient as high risk at admission, these patterns may draw clinical attention to signals of hemodynamic instability, systemic inflammatory burden reflected in the contributing variables. Rather than prescribing specific treatment actions, such explanation outputs may support situational awareness and encourage preventive measures, such as closer monitoring of renal function, reassessment of nephrotoxic medications, optimization of hemodynamic status, or consideration of early nephrology consultation.

Consistent with this interpretation framework, we observed heterogeneity in model-attributed risk patterns across baseline eGFR subgroups, suggesting that the model may distribute predicted risk differently across patients with varying kidney function. Patients with lower baseline eGFR showed higher SHAP-attributed contributions from acute hemodynamic stress-related variables compared with those with higher eGFR. These findings do not imply a mechanistic interaction but rather indicate that the model captured non-linear statistical associations between kidney function and acute physiological stress markers. Similar subgroups heterogeneity has been described in prior explainable machine-learning analyses in critical care settings [24–27]. However, such patterns remain observational and hypothesis-generating. Prospective studies incorporating richer causal modeling or interventional designs would be required to determine whether these associations reflect modifiable biological pathways. Importantly, eGFR in this study reflects patients’ physiological status at hospital entry rather than a longitudinal measure of renal function. The higher admission eGFR observed in the AKI group may partly relate to the relative increase criterion embedded in the KDIGO definition, whereby individuals with lower initial creatinine values are more likely to meet a proportional rise threshold during hospitalization.

External validation in MIMIC-IV revealed a reduction in discrimination compared with internal testing, consistent with expected performance attenuation under cross-institutional dataset shift [9]. To examine the influence of missing-data filtering on external performance estimates, we conducted sensitivity analyses across three representative row-level missingness thresholds (0, ≤ 4, and ≤ 7 missing features). These thresholds substantially altered cohort size and case mix, with remaining sample sizes ranging from 40,960 to 3,850 patients and AKI incidence varying from 27.2% to 51.2%. In addition, differences in baseline serum creatinine distributions between cohorts likely reflect underlying population heterogeneity, including variations in healthcare setting, illness severity, and laboratory monitoring practices between the internal hospital cohort and the ICU-enriched MIMIC-IV population. Despite marked shifts in disease prevalence and population composition, changes in model discrimination across thresholds were small. This suggests that the observed AUROC estimates were relatively robust to reasonable variations in data completeness criteria. However, the substantial change in AKI incidence across thresholds indicates that stricter filtering enriched for higher-acuity patients with more complete laboratory documentation. Such shifts may introduce spectrum effects and limit direct comparability across filtered cohorts [28]. Therefore, while sensitivity analyses support stability of discrimination under varying completeness assumptions, external validation should still be interpreted within the context of altered population structure. Prior studies have demonstrated that predictive models may exhibit clinically meaningful variability under dataset shift across institutions and time periods [9, 29]. Prospective validation in less restricted cohorts and evaluation of recalibration strategies remain necessary before routine clinical implementation.

From a clinical perspective, admission-based risk stratification may support early situational awareness rather than definitive diagnostic decision-making. Because the present model relies exclusively on routinely available pre-admission variables, its potential utility lies in identifying patients who may warrant closer monitoring, medication review, or early nephrology consultation at the time of hospital entry. For example, patients classified into lower baseline eGFR subgroups and concurrently exhibiting acute physiological stress markers were assigned higher predicted risk by the model. These findings reflect model-derived risk stratification and can be interpreted as supporting heightened risk awareness. Importantly, predictive models are most likely to influence care when integrated into clinical workflows as early warning or decision-support tools rather than as autonomous decision makers. For instance, a prospective multicenter evaluation of the Targeted Real-time Early Warning System (TREWS) demonstrated that machine-learning–based alerts could help clinicians identify high-risk patients earlier and prioritize timely intervention within routine care processes [30]. Such systems emphasize clinician interpretation and workflow integration rather than relying solely on algorithmic outputs. It is also essential to emphasize that high retrospective discrimination does not by itself establish clinical effectiveness. Prior studies have demonstrated that prediction models with strong internal performance may experience meaningful variability when implemented in real-world workflows, particularly in the presence of dataset shift or evolving practice patterns [9, 29]. Therefore, prospective validation and evaluation of downstream clinical impact—including whether model-informed decision support alters management or improves patient-centered outcomes—are necessary before routine clinical integration [31, 32]. At present, our findings should be regarded as supporting early risk awareness and hypothesis generation rather than immediate deployment.

Several limitations warrant consideration. First, AKI was defined based on changes in serum creatinine relative to the admission baseline. We selected admission SCr to ensure strict temporal separation between predictors and outcomes and to maintain consistent labeling across internal and external cohorts. However, admission creatinine may not accurately reflect true pre-morbid renal function in all patients. Consequently, some degree of misclassification cannot be excluded. The urine output criterion of KDIGO was not incorporated due to inconsistent availability and documentation across datasets, which may have led to underestimation of oliguric AKI. Second, the prediction horizon encompassed AKI occurring at any time during hospitalization, reflecting baseline susceptibility estimation rather than fixed short-term event forecasting. Time-to-event modeling and dynamic risk updating were beyond the scope of the present design and require longitudinal data integration. Third, pharmacological exposures including renin–angiotensin–aldosterone system inhibitors, sodium–glucose cotransporter-2 inhibitors, diuretics, nonsteroidal anti-inflammatory drugs, vasopressors, and nephrotoxic antibiotics were not explicitly modeled. As a result, the current framework primarily captures baseline clinical vulnerability rather than treatment-related or exposure-driven risk components. Incorporating structured medication data and time-aligned exposure modeling would enhance interpretability and potentially improve clinical relevance. Fourth, although sensitivity analyses suggested stable discrimination across varying missingness thresholds in external validation, substantial differences in cohort size and AKI prevalence indicate underlying case-mix variation. Therefore, external performance should not be interpreted as definitive evidence of universal transportability. Finally, although decision curve analysis suggested potential clinical value across relevant risk thresholds, these findings reflect theoretical benefit within retrospective data. Prospective studies are needed to determine whether integrating the model into clinical workflows improves patient outcomes.

## Supplementary Information

Below is the link to the electronic supplementary material.

Supplementary Material 1

## Data Availability

The data underlying this article will be shared upon reasonable request to the corresponding author.

## Abbreviations

AKI: Acute Kidney Injury
AST: Aspartate Aminotransferase
AUROC: Area Under the Receiver Operating Characteristic Curve
BMI: Body Mass Index
CKD: Chronic Kidney Disease
eGFR: Estimated Glomerular Filtration Rate
LOS: Length of Stay
MODS: Multiple Organ Dysfunction Syndrome
Neu%: Neutrophil Percentage
SCr: Serum Creatinine
TBIL: Total Bilirubin
UA: Uric Acid
