# Supplementary material for: Development and external validation of an interpretable early prediction model for acute kidney injury using TabPFN and routine admission data: a retrospective cohort study
Source: BMC Med Inform Decis Mak. 2026 May 12;26:242. doi: 10.1186/s12911-026-03561-7 (PMC13335356; doi:10.1186/s12911-026-03561-7)
Supplement: Supplementary file 1 — Supplementary Material 1 [file 12911_2026_3561_MOESM1_ESM.docx]

**Supplementary Material**

Supplementary Figure S1. Interaction between baseline renal function, clinical stressors, and AKI risk.

Supplementary Figure S2. SHAP value distributions for four key features

Supplementary Figure S3. Performance comparison between TabPFN and a simplified baseline LR model using the same set of routinely available admission variables.

Supplementary Figure S4. Model stability and sensitivity analysis of TabPFN for AKI prediction.

Supplementary Figure S5 External validation performance under different row-missing thresholds.

Supplementary Table S1: Baseline characteristics of current cohort.

Supplementary Table S2: Performance of the TabPFN model under varying sample sizes and train/test split ratios.

Supplementary Table S3 Baseline characteristics of the MIMIC-IV cohort (n = 3,850; ≤4 missing-feature threshold).

Supplementary Table S4 Baseline characteristics of the MIMIC-IV cohort (n = 40,960; no missing-feature threshold).

Supplementary Table S5 Baseline characteristics of the MIMIC-IV cohort (n = 18,788; ≤7 missing-feature threshold).

Supplementary Table S6. Calibration and Probability Estimation Performance of All Models

Supplementary Table S7. Sample Size, AKI Incidence, and Feature Availability Under Different Missingness Thresholds

**
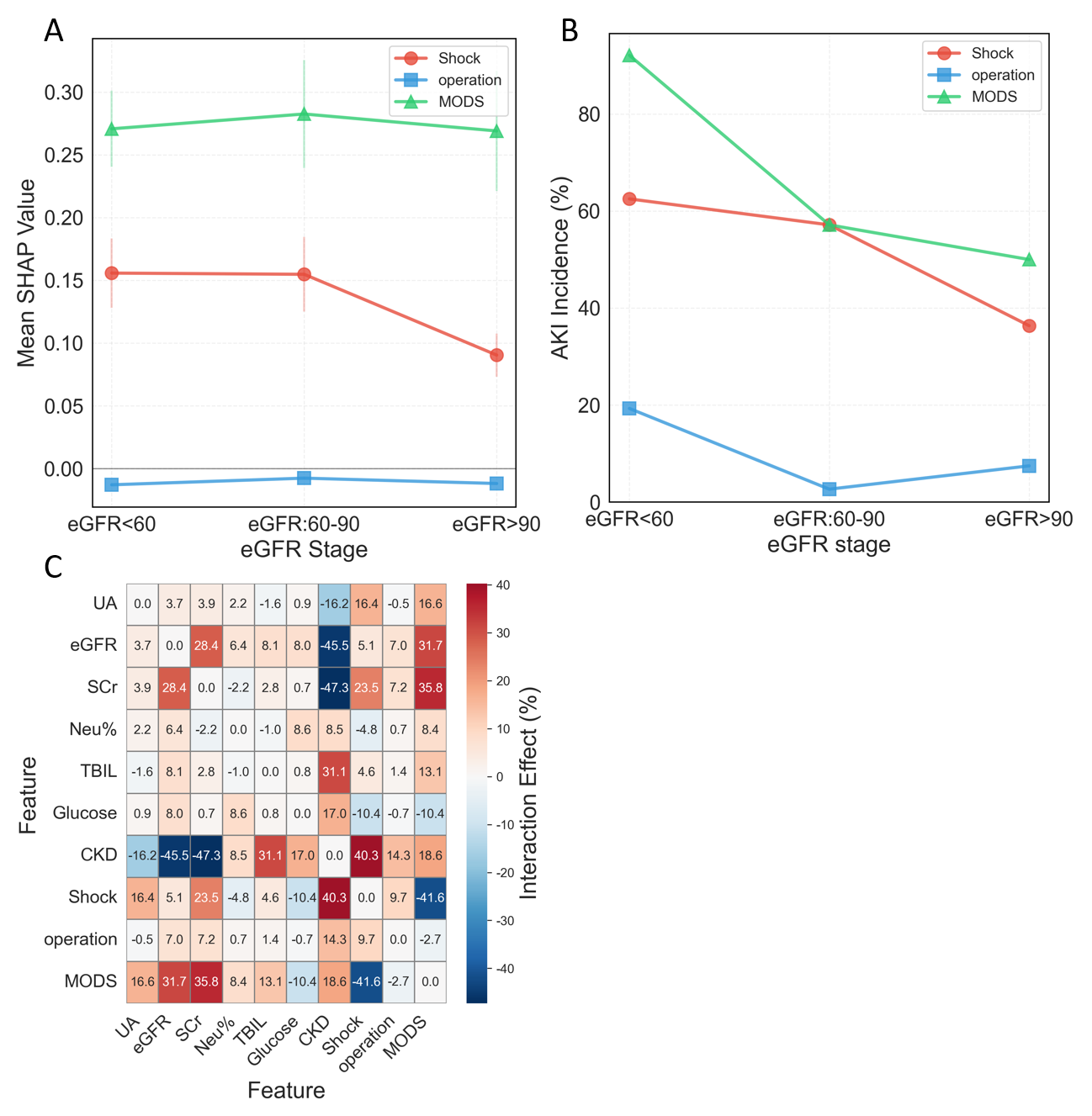
**

**Supplementary Figure S1. Interaction between baseline renal function, clinical stressors, and AKI risk.**

(A) Mean SHAP values across eGFR stages under different stress conditions. (B) Observed AKI incidence across eGFR stages and clinical stressors. (C) Feature–feature interaction effects expressed as percentage contribution.


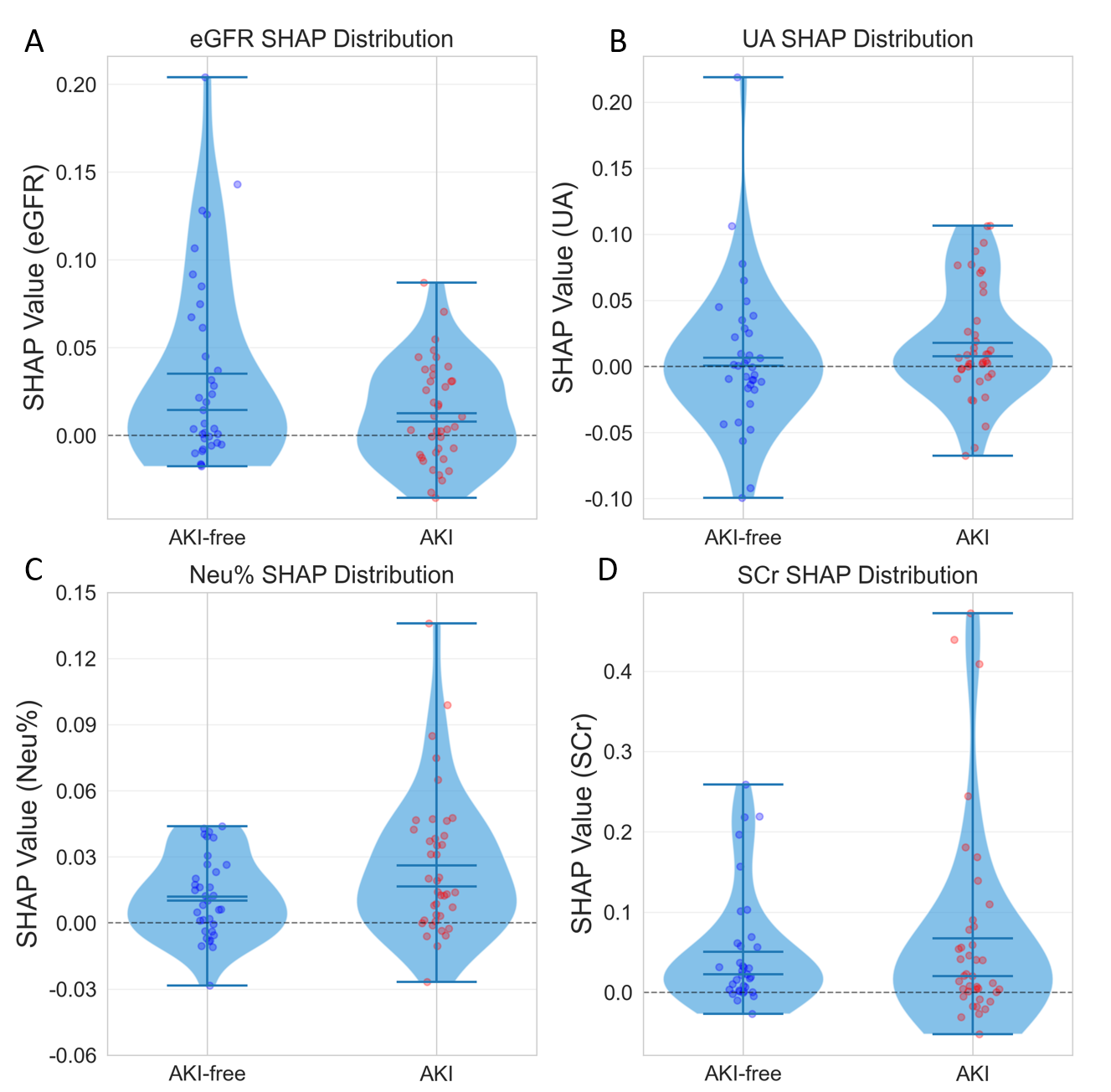


**Supplementary Figure S2. SHAP value distributions for four key features**

1. SHAP value distribution for eGFR. (B) SHAP value distribution for UA. (C) SHAP value distribution for Neu%.(D) SHAP value distribution for SCr.


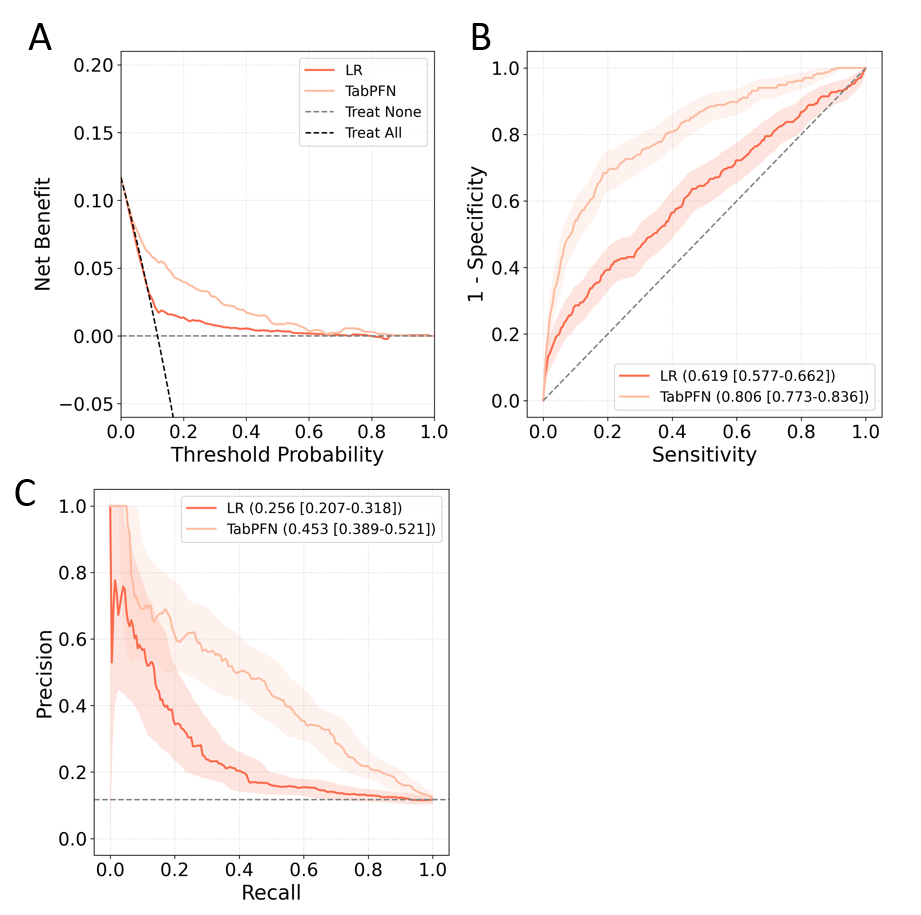


**Supplementary Figure S3. Performance comparison between TabPFN and a simplified baseline LR model using the same set of routinely available admission variables.**

(A) Decision curve analysis showing net benefit across threshold probabilities. (B) ROC curves with corresponding AUROC values. (C) Precision–recall curves with corresponding AUPRC values.


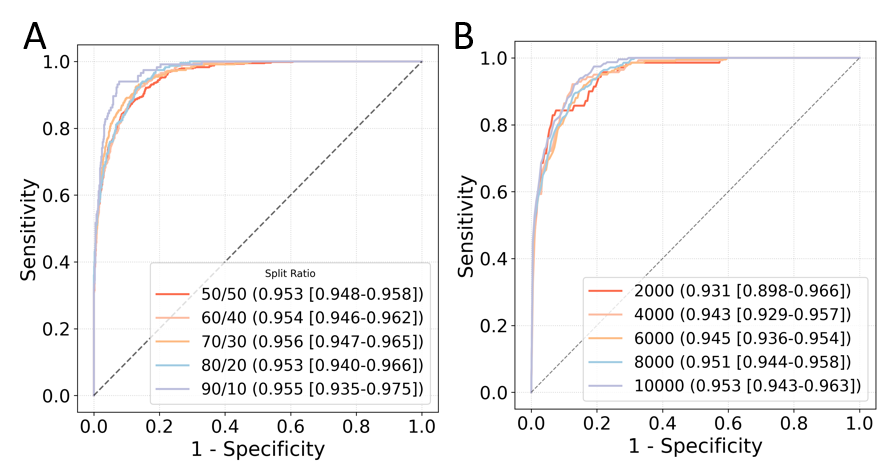


**Supplementary Figure S4.** **Model stability and sensitivity analysis of TabPFN for AKI prediction.**

(A–B) ROC curves under different train-test split ratios and training sample sizes with 95% CI.


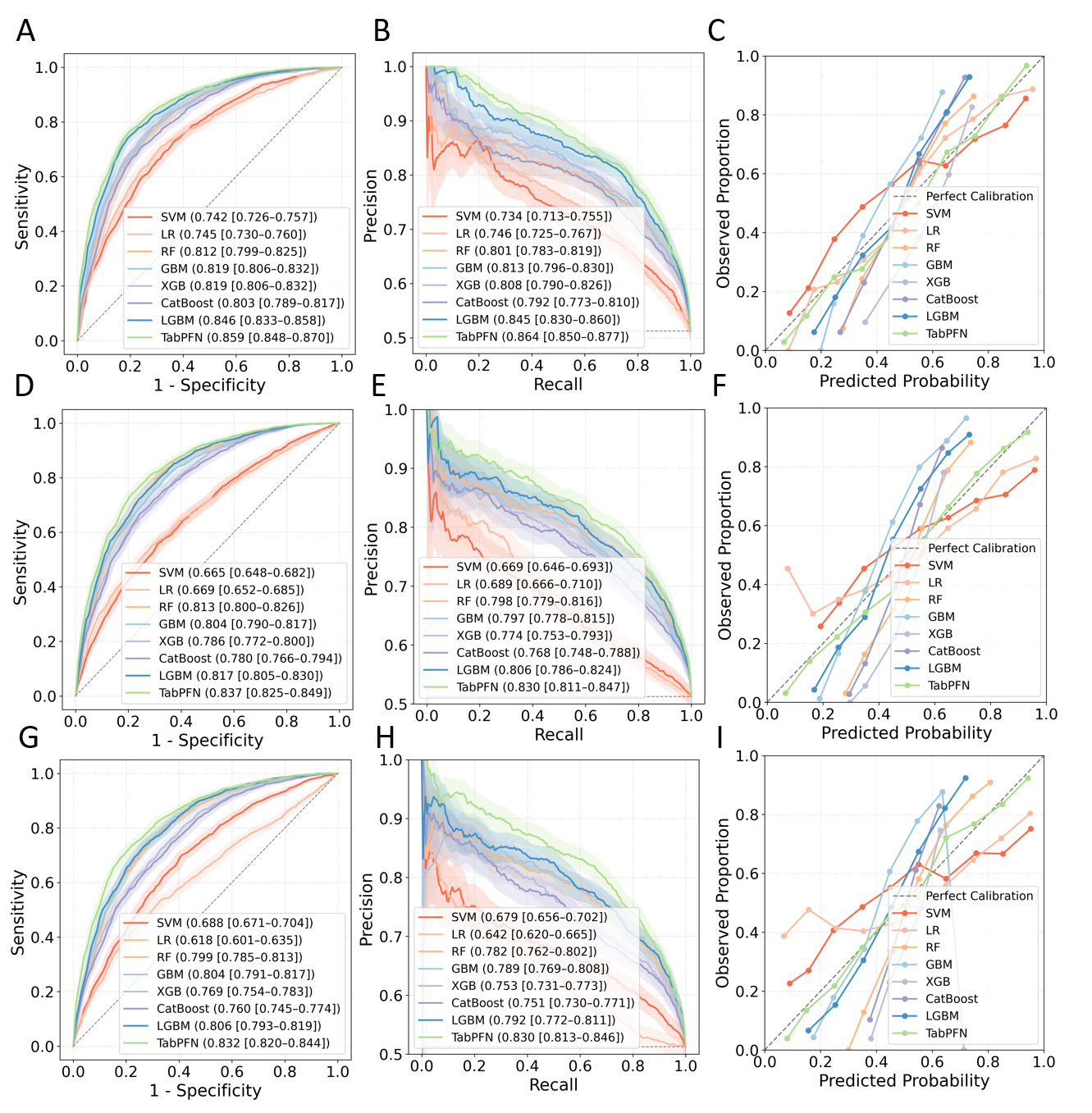


**Supplementary Figure S5. External validation performance under different row-missing thresholds.**

The first row (A–C) corresponds to the ≤4-missing-feature threshold; the second row (D–F) corresponds to the ≤7-missing-feature threshold; and the third row (G–I) corresponds to no row filtering (threshold = 0).
For each threshold, receiver operating characteristic (ROC) curves (left column), precision–recall (PR) curves (middle column), and calibration plots (right column) are shown for all evaluated models.

**Supplementary Table S1. Baseline characteristics of current cohort.**

| **Variable** | | | **AKI-free** | **AKI** | | | **\|t/Z/χ²\|** | | | **p-value** | | |  |
| --- | --- | --- | --- | --- | --- | --- | --- | --- | --- | --- | --- | --- | --- |
| Demographics | | |  |  | | |  | | |  | | |  |
| Male (n/%) | | | 23434 (59.8%) | 2953 (57.1%) | | | 13.782 | | | <0.001 | | |  |
| Age (year) | | | 63 (52-72) | 63 (49-73) | | | 2.318 | | | 0.020 | | |  |
| Smoke (n/%) | | | 13568 (34.7%) | 1593 (30.8%) | | | 29.539 | | | <0.001 | | |  |
| Drink (n/%) | | | 10948 (28.0%) | 1310 (25.3%) | | | 15.434 | | | <0.001 | | |  |
| BMI (kg/m²) | | | 24.20 (21.60-26.10) | 24.20 (21.50-26.60) | | | 3.496 | | | <0.001 | | |  |
| Operation (n/%) | | | 14414 (36.8%) | 1225 (23.7%) | | | 342.968 | | | <0.001 | | |  |
| SBP (mmHg) | | | 129 (116-141) | 130 (115-146) | | | 4.312 | | | <0.001 | | |  |
| DBP (mmHg) | | | 78 (70-85) | 78 (68-87) | | | 0.432 | | | 0.665 | | |  |
|  | | |  |  | | |  | | |  | | |  |
| Laboratory tests | | |  |  | | |  | | |  | | |  |
| SCr (μmol/L) | | | 80.00 (66.20-94.00) | 69.00 (46.18-111.45) | | | 15.915 | | | <0.001 | | |  |
| Glucose (mmol/L) | | | 5.30 (4.70-6.60) | 6.30 (5.00-8.60) | | | 28.801 | | | <0.001 | | |  |
| Na (mmol/L) | | | 141.00 (138.20-143.00) | 139.00 (135.60-142.00) | | | 27.484 | | | <0.001 | | |  |
| Baso (10⁹/L) | | | 0.02 (0.01-0.03) | 0.02 (0.01-0.03) | | | <0.001 | | | — | | |  |
| Hb (g/L) | | | 126.00 (107.00-140.00) | 120.00 (99.00-137.00) | | | 13.485 | | | <0.001 | | |  |
| Eos% (%) | | | 1.50 (0.60-2.70) | 0.70 (0.10-1.80) | | | 34.293 | | | <0.001 | | |  |
| MPV (fL) | | | 9.80 (9.10-10.50) | 9.90 (9.10-10.70) | | | 6.541 | | | <0.001 | | |  |
| PLT (10⁹/L) | | | 219.00 (168.00-274.00) | 192.00 (134.75-250.00) | | | 21.394 | | | <0.001 | | |  |
| Neu% (%) | | | 62.80 (53.70-73.10) | 74.30 (61.90-84.90) | | | 41.874 | | | <0.001 | | |  |
| MCHC (g/L) | | | 333.00 (324.00-341.00) | 335.00 (325.00-344.00) | | | 9.809 | | | <0.001 | | |  |
| RBC (10¹²/L) | | | 4.20 (3.70-4.60) | 4.00 (3.40-4.50) | | | 15.421 | | | <0.001 | | |  |
| WBC (10⁹/L) | | | 6.40 (5.00-8.50) | 7.90 (5.70-11.50) | | | 28.504 | | | <0.001 | | |  |
| MCV (fL) | | | 90.00 (86.60-93.50) | 89.90 (86.20-93.60) | | | 1.602 | | | 0.109 | | |  |
| MCH (pg) | | | 30.10 (28.70-31.40) | 30.20 (28.80-31.50) | | | 3.160 | | | 0.002 | | |  |
| Mono (10⁹/L) | | | 0.50 (0.30-0.60) | 0.50 (0.40-0.70) | | | 9.009 | | | <0.001 | | |  |
| Neu (10⁹/L) | | | 3.90 (2.80-5.80) | 5.60 (3.50-9.30) | | | 35.593 | | | <0.001 | | |  |
| Eos (10⁹/L) | | | 0.10 (0.00-0.20) | 0.00 (0.00-0.10) | | | 24.923 | | | <0.001 | | |  |
| ALT (U/L) | | | 19.00 (13.00-32.00) | 23.00 (14.00-53.00) | | | 17.922 | | | <0.001 | | |  |
| GGT (U/L) | | | 22.00 (14.00-47.70) | 26.00 (14.00-71.00) | | | 9.317 | | | <0.001 | | |  |
| ADA (U/L) | | | 11.00 (9.00-15.00) | 12.00 (8.00-17.00) | | | 4.484 | | | <0.001 | | |  |
| TBIL (μmol/L) | | | 13.40 (9.40-19.60) | 15.70 (10.10-28.20) | | | 18.197 | | | <0.001 | | |  |
| AST (U/L) | | | 18.70 (14.00-28.00) | 24.00 (16.00-55.00) | | | 27.231 | | | <0.001 | | |  |
| TC (mmol/L) | | | 4.60 (3.70-5.40) | 4.20 (3.30-5.20) | | | 16.148 | | | <0.001 | | |  |
| HDL (mmol/L) | | | 1.20 (1.00-1.40) | 1.10 (0.80-1.40) | | | 20.344 | | | <0.001 | | |  |
| LDH (U/L) | | | 165.00 (138.00-210.00) | 197.00 (156.00-281.65) | | | 30.341 | | | <0.001 | | |  |
| UA (μmol/L) | | | 279.00 (212.00-353.00) | 288.00 (189.00-400.00) | | | 3.762 | | | <0.001 | | |  |
| LDL (mmol/L) | | | 2.60 (2.00-3.20) | 2.30 (1.60-3.00) | | | 17.712 | | | <0.001 | | |  |
| A/G | | | 1.30 (1.10-1.50) | 1.20 (1.00-1.50) | | | 13.416 | | | <0.001 | | |  |
| hdl.ldl | | | 3.80 (3.10-4.60) | 3.50 (2.70-4.30) | | | 20.169 | | | <0.001 | | |  |
| TG (mmol/L) | | | 1.10 (0.80-1.60) | 1.20 (0.80-1.80) | | | 7.389 | | | <0.001 | | |  |
| ALP (U/L) | | | 74.00 (58.00-98.90) | 76.00 (57.00-117.00) | | | 5.318 | | | <0.001 | | |  |
| TP (g/L) | | | 64.20 (58.70-69.40) | 60.40 (53.90-66.50) | | | 27.675 | | | <0.001 | | |  |
| ALB (g/L) | | | 36.40 (31.60-40.20) | 33.40 (28.20-38.00) | | | 27.272 | | | <0.001 | | |  |
| TT Ratio | | | 1.10 (1.00-1.20) | 1.10 (1.00-1.20) | | | 13.155 | | | <0.001 | | |  |
| PT% (%) | | | 115.00 (95.90-137.00) | 102.00 (81.00-125.00) | | | 26.905 | | | <0.001 | | |  |
| APTT (s) | | | 1.20 (1.00-1.30) | 1.20 (1.10-1.30) | | | 10.411 | | | <0.001 | | |  |
| PT (s) | | | 10.60 (9.60-11.90) | 11.50 (10.10-13.30) | | | 28.686 | | | <0.001 | | |  |
| FIB (g/L) | | | 3.30 (2.70-4.00) | 3.30 (2.60-4.10) | | | 1.168 | | | 0.242 | | |  |
| TT (s) | | | 14.70 (13.40-16.60) | 15.30 (13.60-17.50) | | | 13.872 | | | <0.001 | | |  |
| Hct (%) | | | 37.60 (32.40-41.70) | 35.60 (29.30-40.80) | | | 16.090 | | | <0.001 | | |  |
| Baso% (%) | | | 0.30 (0.20-0.50) | 0.20 (0.10-0.40) | | | 19.891 | | | <0.001 | | |  |
| eGFR (mL/min/1.73m²) | | | 82.10 (68.00-102.20) | 98.20 (55.30-147.83) | | | 14.455 | | | <0.001 | | |  |
| Comorbidities | | |  |  | | |  | | |  | | |  |
| CKD (n/%) | | | 1889 (4.8%) | 739 (14.3%) | | | 733.189 | | | <0.001 | | |  |
| NS (n/%) | | | 631 (1.6%) | 24 (0.5%) | | | 40.478 | | | <0.001 | | |  |
| Duplexkidney (n/%) | | | 9 (0.0%) | 0 (0.0%) | | | 0.326 | | | 0.568 | | |  |
| Nephritis (n/%) | | | 717 (1.8%) | 49 (0.9%) | | | 20.443 | | | <0.001 | | |  |
| Renalcalculi (n/%) | | | 1028 (2.6%) | 199 (3.9%) | | | 25.009 | | | <0.001 | | |  |
| Hydronephrosis (n/%) | | | 370 (0.9%) | 97 (1.9%) | | | 37.151 | | | <0.001 | | |  |
| Cystickidneydisease (n/%) | | | 1774 (4.5%) | 201 (3.9%) | | | 4.261 | | | 0.039 | | |  |
| Nephrarctia (n/%) | | | 86 (0.2%) | 30 (0.6%) | | | 21.415 | | | <0.001 | | |  |
| Nephrotuberculosis (n/%) | | | 6 (0.0%) | 1 (0.0%) | | | <0.001 | | | — | | |  |
| RF (n/%) | | | 1338 (3.4%) | 424 (8.2%) | | | 272.842 | | | <0.001 | | |  |
| ARDS (n/%) | | | 390 (1.0%) | 33 (0.6%) | | | 5.800 | | | 0.016 | | |  |
| Pneumonia (n/%) | | | 2656 (6.8%) | 184 (3.6%) | | | 78.534 | | | <0.001 | | |  |
| Bronchitis (n/%) | | | 649 (1.7%) | 54 (1.0%) | | | 10.587 | | | 0.001 | | |  |
| Asthma (n/%) | | | 645 (1.6%) | 63 (1.2%) | | | 5.057 | | | 0.025 | | |  |
| Pulmonaryemphysema (n/%) | | 936 (2.4%) | | | 82 (1.6%) | | | 12.788 | | | <0.001 | | |
| COPD (n/%) | | | 1069 (2.7%) | 128 (2.5%) | | | 1.021 | | | 0.312 | | |  |
| PE (n/%) | | | 355 (0.9%) | 27 (0.5%) | | | 7.443 | | | 0.006 | | |  |
| Pulmonarytuberculosis (n/%) | | 507 (1.3%) | | | 40 (0.8%) | | | 9.738 | | | 0.002 | | |
| Pneumonedema (n/%) | | | 12 (0.0%) | 7 (0.1%) | | | 9.385 | | | 0.002 | | |  |
| PAH (n/%) | | | 834 (2.1%) | 319 (6.2%) | | | 292.898 | | | <0.001 | | |  |
| ICH (n/%) | | | 1054 (2.7%) | 221 (4.3%) | | | 40.463 | | | <0.001 | | |  |
| CI (n/%) | | | 2690 (6.9%) | 360 (7.0%) | | | 0.052 | | | 0.820 | | |  |
| CT (n/%) | | | 336 (0.9%) | 51 (1.0%) | | | 0.732 | | | 0.392 | | |  |
| Epilepsy (n/%) | | | 234 (0.6%) | 49 (0.9%) | | | 8.299 | | | 0.004 | | |  |
| TIA (n/%) | | | 81 (0.2%) | 7 (0.1%) | | | 0.842 | | | 0.359 | | |  |
| Hepaticfailure (n/%) | | | 143 (0.4%) | 145 (2.8%) | | | 417.463 | | | <0.001 | | |  |
| Hepatitis (n/%) | | | 1735 (4.4%) | 258 (5.0%) | | | 3.220 | | | 0.073 | | |  |
| FLD (n/%) | | | 1089 (2.8%) | 119 (2.3%) | | | 3.765 | | | 0.052 | | |  |
| Pancreatitis (n/%) | | | 425 (1.1%) | 89 (1.7%) | | | 15.598 | | | <0.001 | | |  |
| Cholelithiasis (n/%) | | | 2824 (7.2%) | 259 (5.0%) | | | 33.821 | | | <0.001 | | |  |
| PU (n/%) | | | 1981 (5.1%) | 165 (3.2%) | | | 34.119 | | | <0.001 | | |  |
| Hepaticcirrhosis (n/%) | | | 1267 (3.2%) | 285 (5.5%) | | | 69.500 | | | <0.001 | | |  |
| HE (n/%) | | | 69 (0.2%) | 48 (0.9%) | | | 95.378 | | | <0.001 | | |  |
| Carcinomaofpancreas (n/%) | | | 446 (1.1%) | 70 (1.4%) | | | 1.659 | | | 0.198 | | |  |
| Splenomegaly (n/%) | | | 477 (1.2%) | 112 (2.2%) | | | 30.637 | | | <0.001 | | |  |
| Hypoglycemosis (n/%) | | | 60 (0.2%) | 12 (0.2%) | | | 1.302 | | | 0.254 | | |  |
| DM (n/%) | | | 6681 (17.1%) | 1078 (20.9%) | | | 45.308 | | | <0.001 | | |  |
| HUA (n/%) | | | 743 (1.9%) | 95 (1.8%) | | | 0.058 | | | 0.810 | | |  |
| Hypercholesterolemia (n/%) | | | 18 (0.0%) | 1 (0.0%) | | | 0.262 | | | 0.609 | | |  |
| Azotemia (n/%) | | | 427 (1.1%) | 24 (0.5%) | | | 17.154 | | | <0.001 | | |  |
| HL (n/%) | | | 817 (2.1%) | 80 (1.5%) | | | 6.409 | | | 0.011 | | |  |
| Hypoproteinemia (n/%) | | | 1034 (2.6%) | 203 (3.9%) | | | 27.415 | | | <0.001 | | |  |
| Hyperlactacidemia (n/%) | | | 7 (0.0%) | 16 (0.3%) | | | 69.393 | | | <0.001 | | |  |
| Hyperthyroidism (n/%) | | | 146 (0.4%) | 26 (0.5%) | | | 1.680 | | | 0.195 | | |  |
| Cardiacarrhythmia (n/%) | | | 2683 (6.9%) | 599 (11.6%) | | | 148.820 | | | <0.001 | | |  |
| AF (n/%) | | | 496 (1.3%) | 202 (3.9%) | | | 203.890 | | | <0.001 | | |  |
| ACS (n/%) | | | 2642 (6.7%) | 681 (13.2%) | | | 271.244 | | | <0.001 | | |  |
| CHD (n/%) | | | 6716 (17.2%) | 1179 (22.8%) | | | 99.575 | | | <0.001 | | |  |
| Rhabdomyolysis (n/%) | | | 5 (0.0%) | 10 (0.2%) | | | 38.899 | | | <0.001 | | |  |
| Shock (n/%) | | | 274 (0.7%) | 386 (7.5%) | | | 1421.577 | | | <0.001 | | |  |
| HBp (n/%) | | | 12648 (32.3%) | 1924 (37.2%) | | | 50.009 | | | <0.001 | | |  |
| MODS (n/%) | | | 182 (0.5%) | 430 (8.3%) | | | 2063.277 | | | <0.001 | | |  |
| HSP (n/%) | | | 44 (0.1%) | 4 (0.1%) | | | 0.243 | | | 0.622 | | |  |
| SLE (n/%) | | | 292 (0.7%) | 19 (0.4%) | | | 8.832 | | | 0.003 | | |  |
| Pyemia (n/%) | | | 274 (0.7%) | 82 (1.6%) | | | 43.970 | | | <0.001 | | |  |
| Septicemia (n/%) | | | 371 (0.9%) | 48 (0.9%) | | | 0.003 | | | 0.957 | | |  |
| SIRS (n/%) | | | 16 (0.04%) | 2 (0.04%) | | | <0.001 | | | — | | |  |
| Outcomes | | |  |  | | |  | | |  | | |  |
| LOS (day)  Death (n/%) | 17 (14-24)  568 (1.5%) | | | | 13 (7-25)  899 (17.4%) | | | 31.636  3614.562 | | | <0.001  <0.001 | | |

Continuous variables are expressed as mean ± standard deviation or median (interquartile range), and categorical variables as number (percentage). P-values were calculated for comparisons between the AKI and AKI-free groups.* BMI :Body Mass Index, SBP :Systolic Blood Pressure, DBP :Diastolic Blood Pressure, Scr :Serum Creatinine, Na :Sodium, Hb :Hemoglobin, Eos%:Eosinophil Percentage, MPV :Mean Platelet Volume, PLT :Platelet Count, Neu%:Neutrophil Percentage, MCHC :Mean Corpuscular Hemoglobin Concentration, RBC :Red Blood Cell Count, WBC :White Blood Cell Count, MCV :Mean Corpuscular Volume, MCH :Mean Corpuscular Hemoglobin, Mono :Monocyte Count, Neu :Neutrophil Count, Eos :Eosinophil Count, ALT :Alanine Aminotransferase, GGT :Gamma-Glutamyl Transferase, ADA :Adenosine Deaminase, TBIL :Total Bilirubin, AST :Aspartate Aminotransferase, TC :Total Cholesterol, HDL :High-Density Lipoprotein, LDH :Lactate Dehydrogenase, UA :Uric Acid, LDL :Low-Density Lipoprotein, A/G :Albumin/Globulin Ratio, TG :Triglyceride, ALP :Alkaline Phosphatase, TP :Total Protein, ALB :Albumin, PT%:Prothrombin Time Percentage, APTT :Activated Partial Thromboplastin Time, PT :Prothrombin Time, FIB :Fibrinogen, TT :Thrombin Time, Hct :Hematocrit, eGFR :Estimated Glomerular Filtration Rate, NS :Nephrotic Syndrome, RF :Respiratory Failure, ARDS :Acute Respiratory Distress Syndrome, COPD :Chronic Obstructive Pulmonary Disease, PE :Pulmonary Embolism, PAH :Pulmonary Arterial Hypertension, ICH :Intracerebral Hemorrhage, CI :Cerebral Infarction, CT :Cerebral Thrombosis, TIA :Transient Ischemic Attack, FLD :Fatty Liver Disease, PU :Peptic Ulcer, HE :Hepatic Encephalopathy, DM :Diabetes Mellitus, HUA :Hyperuricemia, HL :Hyperlipidemia, AF :Atrial Fibrillation, ACS :Acute Coronary Syndrome, CHD :Coronary Heart Disease, HBp :Hypertension, MODS :Multiple Organ Dysfunction Syndrome, HSP :Henoch–Schönlein Purpura, SLE :Systemic Lupus Erythematosus, SIRS:Systemic Inflammatory Response Syndrome, LOS :Length of Stay.

**Supplementary Table S2. Performance of the TabPFN model under varying sample sizes and train/test split ratios.**

|  | **AUROC** | **F1-score** | **precision** | **recall** | **accuracy** |
| --- | --- | --- | --- | --- | --- |
| **Sample_size** |  |  |  |  |  |
| 2000 | 0.931 (0.898, 0.966) | 0.595 (0.505, 0.686) | 0.776 (0.638, 0.914) | 0.490 (0.260, 0.720) | 0.924 (0.898, 0.950) |
| 4000 | 0.943 (0.929, 0.957) | 0.619 (0.542, 0.696) | 0.779 (0.707, 0.851) | 0.514 (0.420, 0.608) | 0.926 (0.916, 0.936) |
| 6000 | 0.945 (0.936, 0.954) | 0.632 (0.585, 0.679) | 0.789 (0.719, 0.859) | 0.529 (0.440, 0.618) | 0.929 (0.922, 0.936) |
| 8000 | 0.951 (0.944, 0.958) | 0.649 (0.590, 0.708) | 0.794 (0.720, 0.868) | 0.551 (0.470, 0.632) | 0.931 (0.923, 0.939) |
| 10000 | 0.953 (0.948, 0.964) | 0.647 (0.600, 0.740) | 0.807 (0.740, 0.880) | 0.542 (0.450, 0.630) | 0.931 (0.928, 0.934) |
| **Split_ratio** |  |  |  |  |  |
| 50/50 | 0.953 (0.948, 0.958) | 0.665 (0.641, 0.689) | 0.801 (0.730, 0.872) | 0.569 (0.520, 0.618) | 0.933 (0.928, 0.938) |
| 60/40 | 0.954 (0.946, 0.962) | 0.665 (0.630, 0.700) | 0.802 (0.730, 0.874) | 0.570 (0.520, 0.620) | 0.933 (0.926, 0.940) |
| 70/30 | 0.956 (0.947, 0.965) | 0.671 (0.635, 0.707) | 0.809 (0.730, 0.888) | 0.575 (0.525, 0.625) | 0.935 (0.927, 0.943) |
| 80/20 | 0.953 (0.948, 0.964) | 0.647 (0.600, 0.740) | 0.807 (0.740, 0.880) | 0.542 (0.450, 0.630) | 0.931 (0.928, 0.934) |
| 90/10 | 0.955 (0.935, 0.975) | 0.667 (0.600, 0.734) | 0.808 (0.716, 0.900) | 0.570 (0.490, 0.650) | 0.934 (0.923, 0.945) |

AUROC: Area Under the Receiver Operating Characteristic Curve

**Supplementary Table S3 Baseline characteristics of the MIMIC-IV cohort (n = 3,850; ≤4 missing-feature threshold).**

| **Variable** | **AKI-free** | **AKI** | **\|t/Z/χ²\|** | **p-value** |
| --- | --- | --- | --- | --- |
| Demographics |  |  |  |  |
| Age (year) | 65.00 (54-76) | 62.00 (52-72) | 6.155 | <0.001 |
| DBP (mmHg) | 70 (58-82) | 66 (55-79) | 5.607 | <0.001 |
| Male (n/%) | 1058 (56.4%) | 1227 (62.2%) | 13.279 | <0.001 |
| SBP (mmHg) | 126 (109-145) | 120 (104-137) | 7.402 | <0.001 |
| Smoke (n/%) | 233 (12.4%) | 252 (12.8%) | 0.082 | 0.774 |
| Laboratory tests |  |  |  |  |
| ALB (g/L) | 3.50 (3.00-3.90) | 3.00 (2.50-3.40) | 19.497 | <0.001 |
| ALP (U/L) | 75.00 (59.00-101.00) | 85.00 (59.00-126.00) | 6.736 | <0.001 |
| ALT (U/L) | 24.00 (15.00-44.00) | 33.00 (18.00-82.00) | 10.270 | <0.001 |
| AST (U/L) | 31.00 (21.00-61.00) | 50.00 (27.00-147.00) | 15.203 | <0.001 |
| Baso (10⁹/L) | 0.30 (0.10-0.50) | 0.20 (0.00-0.40) | 11.774 | <0.001 |
| Eos (10⁹/L) | 0.70 (0.10-1.70) | 0.40 (0.00-1.30) | 7.706 | <0.001 |
| Glucose (mmol/L) | 124.00 (103.00-160.00) | 135.00 (108.00-186.00) | 6.821 | <0.001 |
| Hb (g/L) | 11.20 (9.50-13.00) | 10.20 (8.30-12.00) | 11.783 | <0.001 |
| Hct (%) | 34.10 (28.80-39.00) | 30.70 (25.70-36.30) | 11.590 | <0.001 |
| LDH (U/L) | 272.65 (206.96-370.13) | 344.00 (246.00-547.00) | 14.415 | <0.001 |
| MCH (pg) | 30.20 (28.60-31.70) | 30.50 (28.90-32.00) | 4.084 | <0.001 |
| MCHC (g/L) | 33.10 (32.10-34.10) | 32.90 (31.80-34.10) | 3.163 | 0.002 |
| MCV (fL) | 91.00 (87.00-95.00) | 92.00 (88.00-97.00) | 7.081 | <0.001 |
| MONO (10⁹/L) | 5.40 (3.60-7.90) | 5.10 (3.00-8.00) | 2.270 | 0.023 |
| Na (mmol/L) | 139.00 (136.00-141.00) | 138.00 (135.00-141.00) | 3.542 | <0.001 |
| Neu% (%) | 78.00 (69.00-85.00) | 81.00 (73.00-87.00) | 7.388 | <0.001 |
| PLT (10⁹/L) | 196.00 (142.00-259.00) | 168.00 (103.00-242.00) | 8.697 | <0.001 |
| PT (s) | 13.60 (12.20-15.90) | 15.30 (13.10-19.30) | 14.656 | <0.001 |
| RBC (10¹²/L) | 3.79 (3.18-4.35) | 3.36 (2.74-4.02) | 12.926 | <0.001 |
| SCr (μmol/L) | 79.56 (61.88-106.08) | 114.92 (79.56-203.32) | 19.463 | <0.001 |
| TBIL (μmol/L) | 10.26 (6.84-15.39) | 13.68 (8.55-37.62) | 12.741 | <0.001 |
| TG (mmol/L) | 113.00 (81.00-163.99) | 133.00 (88.00-214.00) | 8.064 | <0.001 |
| WBC (10⁹/L) | 10.60 (7.90-14.40) | 12.00 (7.90-17.00) | 5.471 | <0.001 |
| eGFR (mL/min/1.73m²) | 79.20 (53.19-95.74) | 51.67 (26.76-84.56) | 17.573 | <0.001 |
| Comorbidities |  |  |  |  |
| ARDS (n/%) | 208 (11.1%) | 520 (26.4%) | 145.361 | <0.001 |
| Asthma (n/%) | 51 (2.7%) | 38 (1.9%) | 2.327 | 0.127 |
| CKD (n/%) | 117 (6.2%) | 250 (12.7%) | 45.483 | <0.001 |
| COPD (n/%) | 13 (0.7%) | 20 (1.0%) | 0.820 | 0.365 |
| DM (n/%) | 256 (13.6%) | 285 (14.4%) | 0.453 | 0.501 |
| Hepaticfailure (n/%) | 235 (12.5%) | 315 (16.0%) | 9.047 | 0.003 |
| Operation (n/%) | 42 (2.2%) | 73 (3.7%) | 6.603 | 0.010 |
| Outcomes |  |  |  |  |
| LOS (day) | 10.00 (6.50-17.20) | 21.20 (12.60-33.70) | 25.156 | <0.001 |

Continuous variables are expressed as mean ± standard deviation or median (interquartile range), and categorical variables as number (percentage). P-values were calculated for comparisons between the AKI and AKI-free groups.* ALB: Albumin; ALP: Alkaline Phosphatase; ALT: Alanine Aminotransferase; ARDS: Acute Respiratory Distress Syndrome; AST: Aspartate Aminotransferase; Baso: Basophils; CKD: Chronic Kidney Disease; COPD: Chronic Obstructive Pulmonary Disease; DBP: Diastolic Blood Pressure; DM: Diabetes Mellitus; Eos: Eosinophils; Hb: Hemoglobin; Hct: Hematocrit; Hepaticfailure: Hepatic Failure; LDH: Lactate Dehydrogenase; LOS: Length of Stay; MCH: Mean Corpuscular Hemoglobin; MCHC: Mean Corpuscular Hemoglobin Concentration; MCV: Mean Corpuscular Volume; MONO: Monocytes; Na: Sodium; Neu%: Neutrophil Percentage; PLT: Platelets; PT: Prothrombin Time; RBC: Red Blood Cells; SBP: Systolic Blood Pressure; Scr: Serum Creatinine; TBIL: Total Bilirubin; TG: Triglycerides; WBC: White Blood Cells; eGFR: Estimated Glomerular Filtration Rate.

**Supplementary Table S4 Baseline characteristics of the MIMIC-IV cohort (n = 40,960; no missing-feature threshold).**

| **Variable** | **AKI-free** | **AKI** | **\|t/Z/χ²\|** | **p-value** |
| --- | --- | --- | --- | --- |
| Demographics |  |  |  |  |
| Age (year) | 65.00(53.00-76.00) | 67.00(56.00-77.00) | 10.658 | <0.001 |
| DBP (mmHg) | 67.00(57.00-79.00) | 64.00(54.00-77.00) | 14.639 | <0.001 |
| Male (n/%) | 16742(56.2%) | 6690(60.0%) | 47.757 | <0.001 |
| SBP (mmHg) | 123.00(108.00-140.00) | 120.00(104.00-138.00) | 12.343 | <0.001 |
| Smoke (n/%) | 5096(17.1%) | 1840(16.5%) | 2.055 | 0.152 |
| Laboratory tests |  |  |  |  |
| Glucose (mmol/L) | 124.00(104.00-156.00) | 132.00(106.00-172.00) | 14.189 | <0.001 |
| Hb (g/L) | 10.80(9.30-12.40) | 10.00(8.50-11.70) | 26.617 | <0.001 |
| Hct (%) | 32.50(28.10-37.20) | 30.60(26.20-35.70) | 22.813 | <0.001 |
| MCH (pg) | 30.30(28.80-31.60) | 30.30(28.80-31.70) | 1.093 | 0.274 |
| MCHC (g/L) | 33.20(32.20-34.20) | 32.90(31.70-34.00) | 20.177 | <0.001 |
| MCV (fL) | 91.00(87.00-95.00) | 92.00(88.00-96.00) | 14.921 | <0.001 |
| Na (mmol/L) | 139.00(136.00-141.00) | 139.00(135.00-141.00) | 5.109 | <0.001 |
| PLT (10⁹/L) | 193.00(142.00-256.00) | 176.00(121.00-246.00) | 17.027 | <0.001 |
| PT (s) | 13.80(12.30-15.70) | 14.70(12.90-17.90) | 30.279 | <0.001 |
| RBC (10¹²/L) | 3.60(3.09-4.14) | 3.34(2.83-3.95) | 25.838 | <0.001 |
| SCr (μmol/L) | 79.56(61.88-106.08) | 106.08(70.72-185.64) | 50.046 | <0.001 |
| WBC (10⁹/L) | 10.70(7.80-14.50) | 11.50(8.00-16.00) | 12.420 | <0.001 |
| eGFR (mL/min/1.73m²) | 83.25(56.87-99.16) | 56.42(28.88-86.97) | 50.945 | <0.001 |
| Comorbidities |  |  |  |  |
| ARDS (n/%) | 2694(9.0%) | 2380(21.3%) | 1129.807 | <0.001 |
| Asthma (n/%) | 1002(3.4%) | 326(2.9%) | 4.857 | 0.028 |
| CKD (n/%) | 2412(8.1%) | 2036(18.3%) | 864.409 | <0.001 |
| COPD (n/%) | 395(1.3%) | 193(1.7%) | 9.120 | 0.003 |
| DM (n/%) | 4767(16.0%) | 2293(20.6%) | 118.092 | <0.001 |
| Hepaticfailure (n/%) | 3725(12.5%) | 2310(20.7%) | 434.882 | <0.001 |
| Outcomes |  |  |  |  |
| LOS (day) | 6.80(4.60-10.50) | 11.80(7.00-20.00) | 63.049 | <0.001 |

**Supplementary Table S5 Baseline characteristics of the MIMIC-IV cohort (n = 18,788; ≤7 missing-feature threshold).**

| **Variable** | **AKI-free** | **AKI** | **\|t/Z/χ²\|** | **p-value** |
| --- | --- | --- | --- | --- |
| Demographics |  |  |  |  |
| Age (year) | 65.00(53.00-76.00) | 66.00(55.00-76.00) | 4.247 | <0.001 |
| DBP (mmHg) | 66.00(56.00-78.00) | 64.00(54.00-77.00) | 7.794 | <0.001 |
| Male (n/%) | 6496(56.5%) | 4370(59.9%) | 20.162 | <0.001 |
| SBP (mmHg) | 121.00(106.00-138.00) | 119.00(103.00-136.00) | 6.473 | <0.001 |
| Smoke (n/%) | 1751(15.2%) | 1148(15.7%) | 0.776 | 0.378 |
| Laboratory tests |  |  |  |  |
| ALB (g/L) | 3.30(2.80-3.76) | 3.10(2.60-3.50) | 21.864 | <0.001 |
| ALP (U/L) | 77.00(59.00-109.00) | 84.00(60.00-125.00) | 8.683 | <0.001 |
| ALT (U/L) | 25.00(15.00-47.00) | 29.00(17.00-65.00) | 11.218 | <0.001 |
| AST (U/L) | 32.00(21.00-61.00) | 43.00(24.00-107.00) | 21.083 | <0.001 |
| Baso (10⁹/L) | 0.20(0.10-0.40) | 0.20(0.00-0.31) | 13.563 | <0.001 |
| Eos (10⁹/L) | 0.70(0.10-1.60) | 0.50(0.00-1.50) | 8.191 | <0.001 |
| Glucose (mmol/L) | 123.00(102.00-155.00) | 132.00(106.00-175.00) | 12.829 | <0.001 |
| Hb (g/L) | 10.50(8.90-12.20) | 9.90(8.40-11.70) | 13.023 | <0.001 |
| Hct (%) | 31.60(27.20-36.60) | 30.40(25.90-35.60) | 10.820 | <0.001 |
| LDH (U/L) | 261.16(200.00-348.55) | 313.00(232.00-468.00) | 27.377 | <0.001 |
| MCH (pg) | 30.20(28.70-31.70) | 30.30(28.70-31.80) | 2.408 | 0.016 |
| MCHC (g/L) | 33.10(32.00-34.10) | 32.80(31.60-33.90) | 11.778 | <0.001 |
| MCV (fL) | 91.00(87.00-95.00) | 92.00(88.00-97.00) | 10.739 | <0.001 |
| MONO (10⁹/L) | 5.00(3.20-7.10) | 5.00(3.00-7.20) | 3.317 | <0.001 |
| Na (mmol/L) | 139.00(136.00-141.00) | 138.00(135.00-141.00) | 1.910 | 0.056 |
| Neu% (%) | 79.00(71.10-85.50) | 81.00(73.60-87.00) | 10.955 | <0.001 |
| PLT (10⁹/L) | 185.00(129.00-257.00) | 173.00(115.00-249.00) | 8.166 | <0.001 |
| PT (s) | 14.20(12.60-16.40) | 15.10(13.10-18.70) | 19.429 | <0.001 |
| RBC (10¹²/L) | 3.50(2.98-4.07) | 3.31(2.79-3.94) | 13.440 | <0.001 |
| SCr (μmol/L) | 79.56(61.88-114.92) | 114.92(70.72-194.48) | 35.834 | <0.001 |
| TBIL (μmol/L) | 10.26(6.84-18.81) | 11.97(6.84-25.65) | 12.916 | <0.001 |
| WBC (10⁹/L) | 10.90(7.70-15.30) | 11.90(8.10-16.80) | 8.871 | <0.001 |
| eGFR (mL/min/1.73m²) | 79.83(51.86-98.47) | 53.85(27.86-85.76) | 35.247 | <0.001 |
| Comorbidities |  |  |  |  |
| ARDS (n/%) | 1387(12.1%) | 1839(25.2%) | 539.521 | <0.001 |
| Asthma (n/%) | 334(2.9%) | 192(2.6%) | 1.156 | 0.282 |
| CKD (n/%) | 915(8.0%) | 1217(16.7%) | 335.679 | <0.001 |
| COPD (n/%) | 134(1.2%) | 128(1.8%) | 10.774 | 0.001 |
| DM (n/%) | 1674(14.6%) | 1353(18.5%) | 51.660 | <0.001 |
| Hepaticfailure (n/%) | 1462(12.7%) | 1413(19.4%) | 151.031 | <0.001 |
| Outcomes |  |  |  |  |
| LOS (day) | 8.90(5.90-14.30) | 14.80(8.90-24.00) | 41.803 | <0.001 |

**Supplementary Table S6. Calibration and Probability Estimation Performance of All Models**

| **Model** | **Brier** | **LogLoss** | **Calibration Intercept** | **Calibration Slope** | **ECE** | **MCE** |
| --- | --- | --- | --- | --- | --- | --- |
| TabPFN | 0.040 (0.035–0.044) | 0.128 (0.116–0.141) | 0.019 (−0.174–0.227) | 1.022 (0.950–1.110) | 0.011 | 0.137 |
| LightGBM | 0.052 (0.047–0.057) | 0.189 (0.176–0.203) | 1.629 (1.391–1.926) | 1.933 (1.819–2.081) | 0.054 | 0.319 |
| GBM | 0.054 (0.048–0.059) | 0.203 (0.189–0.218) | 1.703 (1.408–2.059) | 1.934 (1.794–2.101) | 0.060 | 0.262 |
| RF | 0.055 (0.050–0.060) | 0.203 (0.189–0.218) | 1.626 (1.387–1.891) | 1.978 (1.856–2.121) | 0.056 | 0.349 |
| CatBoost | 0.059 (0.055–0.064) | 0.239 (0.228–0.251) | 2.117 (1.643–2.684) | 2.763 (2.496–3.093) | 0.099 | 0.301 |
| XGBoost | 0.063 (0.059–0.068) | 0.244 (0.234–0.255) | 1.445 (1.235–1.683) | 2.618 (2.463–2.807) | 0.100 | 0.380 |
| LR | 0.079 (0.073–0.085) | 0.288 (0.261–0.319) | −0.525 (−0.851–−0.200) | 0.725 (0.569–0.871) | 0.019 | 0.363 |
| SVM | 0.088 (0.080–0.095) | 0.312 (0.292–0.333) | 0.152 (−0.316–0.761) | 1.102 (0.886–1.383) | 0.073 | 0.298 |

ECE: Expected Calibration Error, MCE: Maximum Calibration Error

**Supplementary Table S7. Sample Size, AKI Incidence, and Feature Availability Under Different Missingness Thresholds**

| **Missing_threshold** | **Remaining_samples** | **AKI_incidence** | **Features** |
| --- | --- | --- | --- |
| 0 | 40960 | 0.272 | 24 |
| 3 | 2451 | 0.428 | 37 |
| 4 | 3850 | 0.512 | 36 |
| 5 | 8973 | 0.488 | 36 |
| 6 | 14562 | 0.434 | 34 |
| 7 | 18788 | 0.388 | 34 |
